# Laboratory-derived inflammatory ratios as a novel diagnostic model for premature coronary artery disease

**DOI:** 10.3389/fendo.2025.1646944

**Published:** 2025-08-27

**Authors:** Fujia Guo, Min Xu, Qingxian Tu, Heyun Yang, Keqiang Linghu, Botao Li, Jie Zhang, Ya Luo, Hong Huang

**Affiliations:** Cardiovascular Medicine, The Third Affiliated Hospital of Zunyi Medical University (The First People’s Hospital of Zunyi), Zunyi Medical University, Zunyi, China

**Keywords:** premature coronary artery disease, monocyte-to-high-density lipoprotein cholesterol ratio, lymphocyte-to-monocyte ratio, apolipoprotein B to apolipoprotein A-1 ratio, platelet-to-lymphocyte ratio, neutrophil-to-lymphocyte ratio, Low-density lipoprotein cholesterol to highdensity lipoprotein cholesterol ratio

## Abstract

**Introduction:**

Coronary artery disease (CAD) is showing a trend toward earlier onset. Premature CAD (PCAD) is clinically defined as CAD with onset before the age of 55 in males and 65 in females. Notably, many young patients subsequently hospitalized with acute cardiovascular events had undergone annual physical examinations before hospitalization, yet were not identified as high-risk by current risk stratification guidelines or traditional risk assessment tools. This study aims to investigate the diagnostic capacity of novel inflammatory biomarkers (including the monocyte-to-high-density lipoprotein cholesterol ratio (MHR), platelet-to-lymphocyte ratio (PLR), neutrophil-to-lymphocyte ratio (NLR), lymphocyte-to-monocyte ratio (LMR), apolipoprotein B to apolipoprotein A-1 ratio (apoB/apoA-1), and low-density lipoprotein cholesterol to high-density lipoprotein cholesterol ratio (LDL-c/HDL-c)) for PCAD, thereby providing the evidence-based foundation for PCAD screening.

**Methods:**

A total of 1,012 young subjects (male<55 years, female<65 years) undergoing diagnostic coronary angiography (CAG) at the Third Affiliated Hospital of Zunyi Medical University (from January 2022 to February 2023) were retrospectively analyzed. We stratified 1,012 eligible participants into two groups: 521 angiographically confirmed PCAD cases and 491 controls with normal coronary arteries. Comprehensive baseline characteristics, including cardiovascular risk profiles and core laboratory-measured inflammatory markers, were recorded. The Mann-Whitney U test and binary logistic regression analysis were employed to assess the associations between inflammatory biomarkers and PCAD. The areas under the receiver operating characteristic (ROC) curves (AUCs) were calculated to evaluate their diagnostic performance for PCAD.

**Results:**

The odds ratio (OR) values for MHR, NLR, LDL-c/HDL-c, and apoB/apoA-1 were 5.592 (95% CI: 2.886-7.836), 1.671 (95% CI: 1.500-1.861), 1.663 (95% CI: 1.419-1.950), and 6.268 (95% CI: 2.765-8.213), respectively (all *P* < 0.001). LMR was not identified as an independent risk factor for PCAD. After adjusting for confounding factors, apoB/apoA-1 remained the strongest risk factor for PCAD compared to other inflammatory markers. The AUCs for MHR, NLR, LDL-c/HDL-c, and apoB/apoA-1 were 0.621 (95% CI: 0.587-0.656), 0.735 (95% CI: 0.703-0.766), 0.605 (95% CI: 0.570-0.640), and 0.771 (95% CI: 0.742-0.799), respectively (all *P* < 0.001). Furthermore, the diagnostic model combining apoB/apoA-1 with neutrophil, lymphocyte, monocyte, triglyceride, uric acid, HDL-c, age, sex, smoking, alcohol consumption, diabetes mellitus, and family history of hypertension, diabetes mellitus, and CAD achieved the highest AUC of 0.898 (95% CI: 0.880-0.917). We analyzed the diagnostic value of inflammatory markers for acute coronary syndrome (ACS) in PCAD patients. The AUCs for these four inflammatory markers were 0.661 (95% CI: 0.626-0.696) for MHR, 0.726 (95% CI: 0.692-0.760) for NLR, 0.615 (95% CI: 0.579-0.651) for LDL-c/HDL-c, and 0.795 (95% CI: 0.766-0.824) for apoB/apoA-1 (all *P* < 0.001), indicating that apoB/apoA-1 had higher diagnostic value for ACS in PCAD than other inflammatory markers. Additionally, the combined diagnostic model incorporating apoB/apoA-1 with the aforementioned covariates achieved an AUC of 0.923 (95% CI: 0.906-0.940) for ACS.

**Conclusions:**

The apoB/apoA-1 outperformed MHR, NLR, and LDL-c/HDL-c as an inflammatory biomarker in PCAD. Its diagnostic capacity was notably enhanced in ACS subgroups. A comprehensive model combining apoB/apoA-1 with traditional risk factors demonstrated exceptional accuracy. Incorporating this biomarker into routine screening protocols could significantly strengthen preventive strategies.

## Introduction

Global Burden of Disease studies indicate that ischemic heart disease remains a primary disease burden across nations and regions. Notably, coronary artery disease (CAD) has emerged as the most prevalent cardiovascular disorder in both developed and developing regions, characterized by high mortality rates and posing a serious threat to public health security (1–3).

An increasing number of studies indicate that the onset of CAD is trending toward younger age groups (4, 5). According to the National Cholesterol Education Program Adult Treatment Panel III guidelines (NCEP-ATP III), premature CAD (PCAD) is defined as CAD occurring in males aged <55 years and females aged <65 years (6). Research has shown that PCAD constitutes 31% of total CAD cases (7). However, compared to patients with mature CAD (MCAD), those with PCAD have a higher incidence of acute coronary syndrome (ACS), as well as increased mortality rates and a poorer long-term prognosis (8–10).

In clinical practice, a phenomenon has been observed: many patients ultimately hospitalized for PCAD report having undergone annual health check-ups before hospitalization, yet none of these examinations included coronary artery screening. The reasons may be that the patients had no symptoms, lacked high-risk factors, or that coronary artery screening is not part of routine physical examinations. Consequently, these patients are often not identified as high-risk by current risk stratification guidelines or traditional risk assessment tools (11, 12), leading to hospitalization for acute cardiovascular events or even death within the subsequent year. Therefore, it is necessary to look for clues in routine health check-ups to guide and assess whether further coronary artery screening is needed, a measure that should be put on the agenda.

Over the past two decades, the conceptualization of CAD-associated atherosclerosis has progressively shifted toward recognition as a chronic, low-grade, lipid-driven inflammatory disorder of the arterial wall. Accumulating evidence indicates that atherosclerosis constitutes a chronic inflammatory arterial pathology, fundamentally mediated by inflammatory mechanisms involving multiple cytokines and biochemical mediators linked to CAD initiation, progression, and clinical outcomes (13–15).

In recent years, numerous diagnostic indicator models for cardiovascular diseases have been proposed, such as the monocyte-to-high-density lipoprotein cholesterol ratio (MHR), lymphocyte-to-monocyte ratio (LMR), neutrophil-to-lymphocyte ratio (NLR), platelet-to- lymphocyte ratio (PLR), apolipoprotein B to apolipoprotein A-1 ratio (apoB/apoA-1), and the low-density lipoprotein cholesterol to high-density lipoprotein cholesterol ratio (LDL-c/HDL-c) (16, 17). These models primarily integrate both protective and risk factors as indicators, leveraging their combined diagnostic power for risk assessment. Compared to individual parameters alone, they exhibit improved diagnostic value through a comprehensive integration of the effects of protective and risk factors.

Some scholars believe that MHR (18–20), LMR (21–23), NLR (24, 25), PLR (26, 27), apoB/apoA-1 (28, 29), and LDL-c/HDL-c (30) have a certain degree of diagnostic effect on CAD, but there is no clear conclusion regarding the diagnostic value of these models for PCAD. This study will explore the diagnostic value of MHR, LMR, NLR, PLR, apoB/apoA-1, and LDL-c/HDL-c for PCAD in order to find reliable diagnostic indicators and provide reference for the screening of PCAD.

## Methods

### Study population

**Figure 1:**
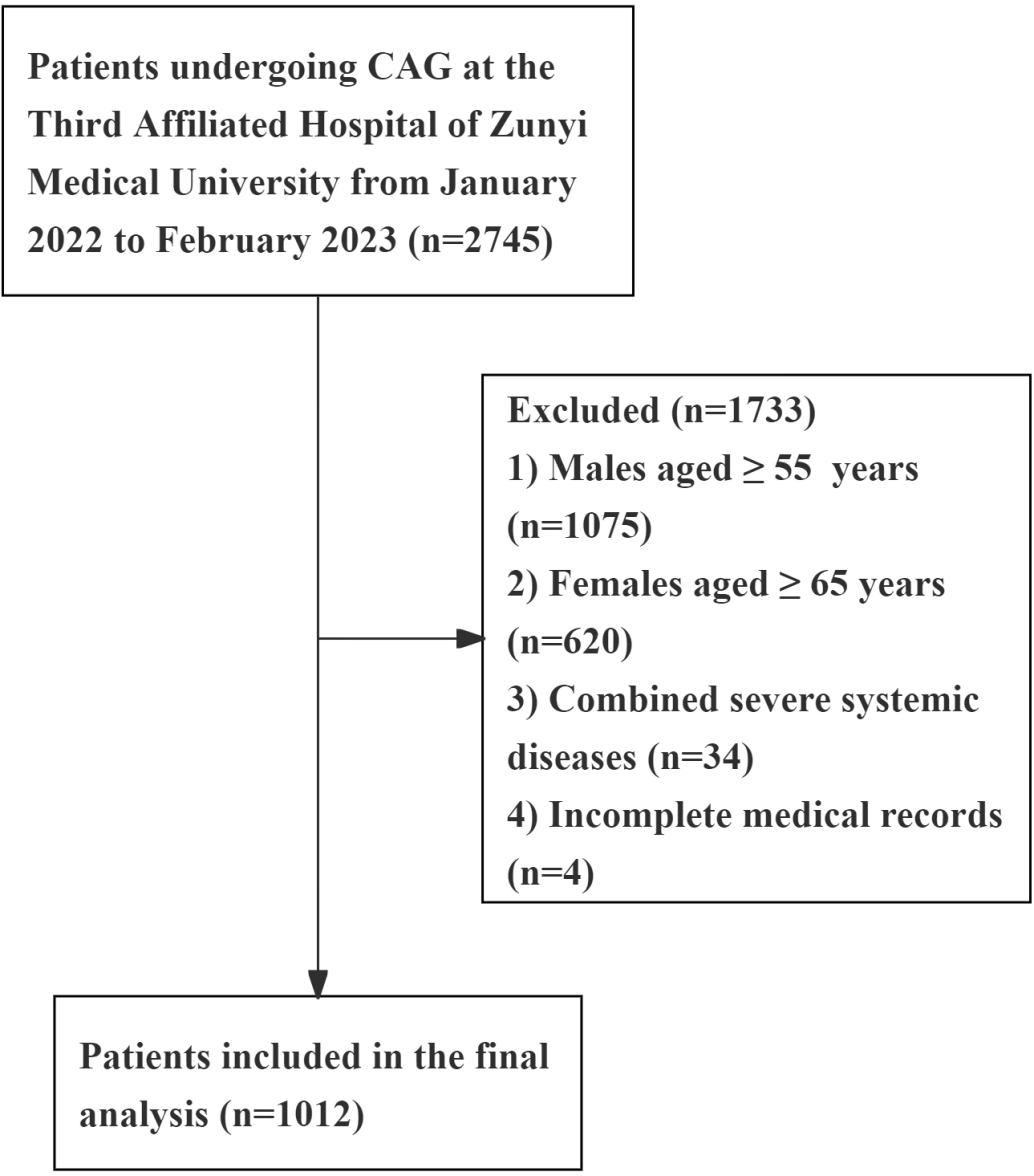
Flow diagram of patient selection. Severe systemic diseases, including: severe infections, hepatic or renal dysfunction, autoimmune diseases, malignancies, myocarditis, pericarditis, severe heart failure, arrhythmias, or connective tissue disorders.

From January 2022 to February 2023, a total of 2,745 patients underwent coronary angiography (CAG) at the Department of Cardiology, Third Affiliated Hospital of Zunyi Medical University. All study subjects were examined using the Judkins method for CAG and diagnosed by at least two cardiovascular interventional specialists according to CAD diagnostic criteria (i.e., ≥50% diameter stenosis in at least one major coronary artery). Based on the NCEP-ATP III guidelines, PCAD was defined as onset occurring before 55 years in males and 65 years in females. Exclusion criteria included severe infections, hepatic or renal dysfunction, autoimmune diseases, malignancies, myocarditis, pericarditis, severe heart failure, arrhythmias, or connective tissue disorders. Ultimately, 1,012 eligible participants were enrolled, comprising 521 PCAD cases and 491 controls with angiographically normal coronary arteries (**Figure 1**).

### Clinical and laboratory data collection

General data included: gender, age, alcohol consumption history (>100 g/day for >1 year), smoking history (≥1 cigarette/day for >1 year), history of hypertension, diabetes mellitus, family history of hypertension, family history of diabetes mellitus, family history of CAD, etc. Biochemical indexes included: neutrophil, lymphocyte, monocyte, serum total cholesterol (TC), triglyceride (TG), high-density lipoprotein cholesterol (HDL-c), low-density lipoprotein cholesterol (LDL-c), blood uric acid (UA), platelet count (PLT), apolipoprotein B, apolipoprotein A-1, etc.

### Definitions

The NLR, PLR and LMR are the absolute value ratios of the corresponding blood cell counts. MHR is calculated as the ratio of the absolute monocyte count divided by the HDL-c, apoB/apoA-1 is the ratio of apoB to apoA-1, LDL-c/HDL-c is the ratio of LDL-c to HDL-c. Diabetes mellitus is diagnosed when fasting plasma glucose is ≥ 7.0 mmol/L, random blood glucose is ≥ 11.1 mmol/L, 2-hour plasma glucose post oral glucose tolerance test is ≥ 11.1 mmol/L, or when an individual is using insulin or oral hypoglycemic agents. Hypertension is defined as a systolic blood pressure ≥ 140 mmHg and/or a diastolic blood pressure ≥ 90 mmHg, or when an individual is on antihypertensive medication.

### Statistical analysis

Statistical analyses were conducted using SPSS software (version 27.0; IBM Corp). Continuous variables were expressed as medians with interquartile ranges, while categorical variables were presented as frequencies and percentages. The Mann-Whitney U test (for non-normally distributed variables) and binary logistic regression analysis were employed to assess associations between inflammatory biomarkers and PCAD. The associations between the inflammatory biomarkers and traditional cardiovascular risk factors were assessed using Spearman correlation analysis. Receiver operating characteristic (ROC) curve analysis evaluated the diagnostic performance of these biomarkers. Statistical significance was defined as *P*<0.05.

## Results

### Clinical, laboratory, and inflammatory characteristics of patients with PCAD

This study enrolled a total of 1,012 eligible participants, comprising 521 patients with PCAD and 491 angiographically normal controls. Anthropometric and laboratory parameters are presented in **Table 1**. Compared with angiographically normal controls, PCAD patients exhibited significantly higher proportions of males, smoking, alcohol consumption, diabetes mellitus, and family histories of hypertension, diabetes mellitus, and CAD (*P* < 0.001), and were significantly younger (*P* < 0.001). No statistically significant difference was observed in hypertension prevalence between the two groups (*P* > 0.05).

**Table 1:** Comparison of clinical, laboratory, and inflammatory characteristics between patients with PCAD and angiographically normal controls.

Variables	PCAD	Angiographically normal controls (N=491)	*P* value
	(N=521)		
Demographics			
Age, years	52 (47-55)	54 (49-60)	<0.001
Male, n (%)	331 (63.5%)	135 (27.5%)	<0.001
Risk factors			
Smoking, n (%)	274 (52.6%)	101 (20.6%)	<0.001
Alcohol consumption, n (%)	116 (22.3%)	46 (9.4%)	<0.001
Medical history			
Hypertension, n (%)	302 (58.0%)	273 (55.6%)	0.448
Diabetes Mellitus, n (%)	82 (15.7%)	24 (4.9%)	<0.001
Family history			
Hypertension, n (%)	75 (14.4%)	21 (4.3%)	<0.001
Diabetes mellitus, n (%)	15 (2.9%)	3 (0.6%)	0.006
CAD, n (%)	28 (5.4%)	7 (1.4%)	<0.001
Laboratory parameters			
Neutrophil (×10^9^/L)	6.20 (4.30-8.00)	3.80 (3.20-4.10)	<0.001
Lymphocyte (×10^9^/L)	1.96 (1.50-2.40)	1.89 (1.60-1.90)	0.01
Monocyte (×10^9^/L)	0.50 (0.33-0.56)	0.45 (0.40-0.50)	<0.001
Platelet (×10^9^/L)	215 (177-257)	209 (176-249)	0.188
UA (μmol/L)	360 (303-397)	317 (266-379)	<0.001
TG (mmol/L)	1.83 (1.27-2.85)	1.55 (1.17-2.41)	<0.001
TC (mmol/L)	4.57 (3.80-5.46)	4.62 (3.91-5.34)	0.759
apoB (mmol/L)	1.03 (0.85-1.15)	0.83 (0.65-0.97)	<0.001
apoA-1 (mmol/L)	1.19 (1.03-1.36)	1.28 (1.14-1.44)	<0.001
LDL-c (mmol/L)	2.73 (2.23-3.31)	2.70 (2.22-3.18)	0.109
HDL-c (mmol/L)	1.05 (0.90-1.29)	1.19 (1.01-1.35)	<0.001
Novel lipid parameters			
apoB/apoA-1	0.85 (0.69-0.95)	0.64 (0.50-0.76)	<0.001
LDL-c/HDL-c	2.58 (1.99-3.22)	2.29 (1.83-2.80)	<0.001
Novel inflammatory parameters			
MHR	0.45 (0.32-0.61)	0.38 (0.29-0.47)	<0.001
LMR	3.67 (3.29-5.25)	4.21 (3.89-4.60)	0.01
NLR	3.08 (2.04-4.22)	2.02 (1.80-2.30)	<0.001
PLR	117.33 (83.39-157.74)	113.33 (92.31-145.98)	0.908

Data were given as median with interquartile range or n (%); CAD, Coronary artery disease; PCAD, Premature CAD; MHR, Monocyte-to-high-density lipoprotein cholesterol ratio; ApoB/apoA-1, Apolipoprotein B to apolipoprotein A-1 ratio; PLR, Platelet-to-lymphocyte ratio; LMR, Lymphocyte-to-monocyte ratio; LDL-c/HDL-c, Low-density lipoprotein cholesterol to high-density lipoprotein cholesterol ratio; NLR, Neutrophil-to-lymphocyte ratio; UA, Uric acid; TG, Triglyceride; TC, Total cholesterol.

Regarding laboratory parameters, compared with angiographically normal controls, the PCAD group demonstrated significantly higher levels of UA, TG, apoB, neutrophil count, lymphocyte count, and monocyte count, whereas significantly lower levels of apoA-1 and HDL-c (*P* < 0.001). No significant differences were observed in PLT, TC, or LDL-c levels between groups (*P* > 0.05).

To further investigate inflammatory and lipid characteristics in PCAD patients, novel biomarkers were evaluated. Inflammatory biomarkers (MHR and NLR) and lipid parameters (apoB/apoA-1 and LDL-c/HDL-c) were significantly elevated in PCAD patients compared to angiographically normal controls (*P* < 0.001). Conversely, LMR was significantly lower in the PCAD group (*P* < 0.001). No statistically significant difference was observed in PLR between groups (*P* > 0.05).

### Association between inflammatory markers and risk of PCAD

**Figure 2:**
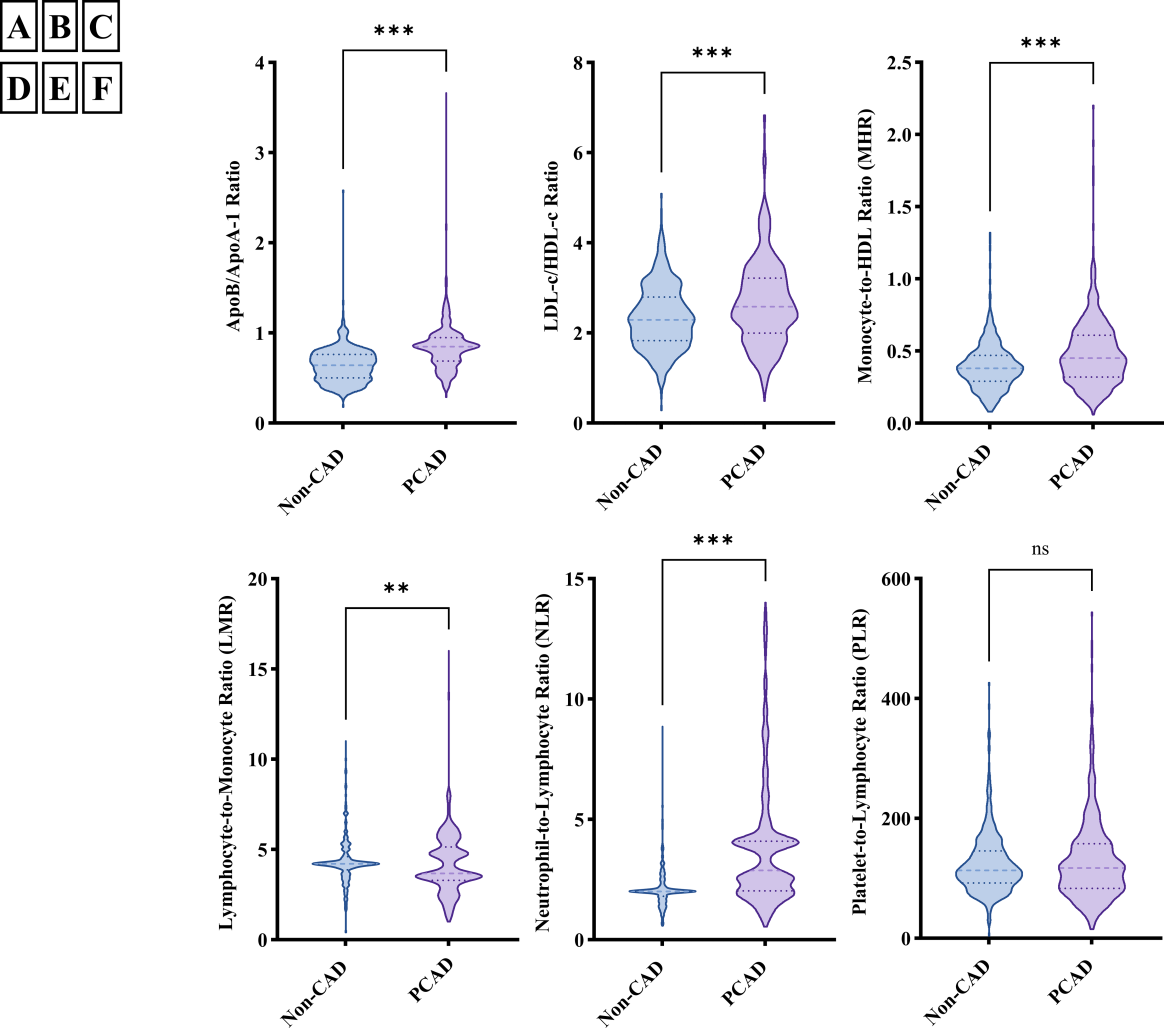
The distribution of inflammatory markers in premature coronary artery disease (PCAD) group and angiographically normal control group; **(A)** ApoB/apoA-1 distribution in PCAD group and angiographically normal control group; **(B)** LDL-c/HDL-c distribution in PCAD group and angiographically normal control group; **(C)** MHR distribution in PCAD group and angiographically normal control group; **(D)** LMR distribution in PCAD group and angiographically normal control group; **(E)** NLR distribution in PCAD group and angiographically normal control group; **(F)** PLR distribution in PCAD group and angiographically normal control group; ApoB/apoA-1, Apolipoprotein B to apolipoprotein A-1 ratio; LDL-c/HDL-c, Low-density lipoprotein cholesterol to high-density lipoprotein cholesterol ratio; MHR, Monocyte-to-high-density lipoprotein cholesterol ratio; LMR, Lymphocyte-to-monocyte ratio; NLR, Neutrophil-to-lymphocyte ratio; PLR, Platelet-to-lymphocyte ratio; ***means *P* < 0.001; **means *P* < 0.05; ns, not significant (*P* > 0.05).

**Figure 3:**
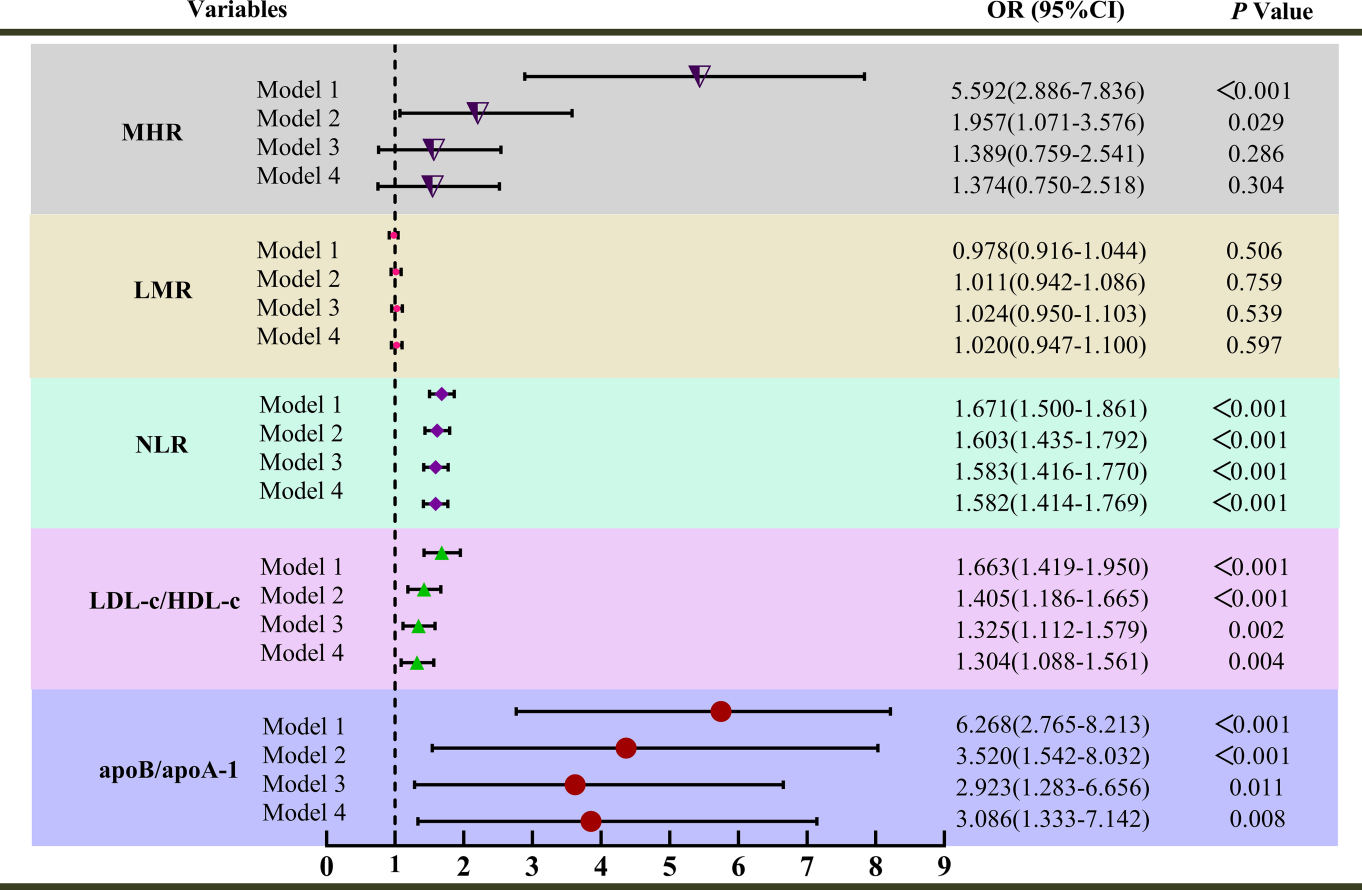
Univariate and multivariate analysis for inflammatory markers associated with premature coronary artery disease (PCAD); Model 1 was unadjusted univariate analysis. Model 2 was adjusted for age, sex. Model 3 was further adjusted for smoking, alcohol consumption, diabetes mellitus, family histories of hypertension, diabetes mellitus, and CAD; Model 4 was further adjusted for uric acid and triglycerides; MHR, Monocyte-to-high-density lipoprotein cholesterol ratio; NLR, Neutrophil-to-lymphocyte ratio; ApoB/apoA-1, Apolipoprotein B to apolipoprotein A-1 ratio; LDL-c/HDL-c, Low-density lipoprotein cholesterol to high-density lipoprotein cholesterol ratio; LMR, Lymphocyte-to-monocyte ratio; OR, Odds ratio.

The distribution of MHR, LMR, NLR, apoB/apoA-1, and LDL-c/HDL-c in the PCAD group and angiographically normal control group is illustrated in **Figure 2**. To explore the association between inflammatory markers and the risk of PCAD, univariate and multivariate logistic regression analyses were performed. As shown in **Figure 3**, MHR, NLR, apoB/apoA-1, and LDL-c/HDL-c were independently associated with the risk of PCAD in both univariate and multivariate analyses. In contrast, LMR was not identified as an independent risk factor for PCAD.

Notably, apoB/apoA-1 exhibited the strongest association with PCAD among the biomarkers in the univariate analysis (Model 1). After adjustment for sex and age (Model 2), the odds ratio (OR) for apoB/apoA-1 was 3.520 (95% CI: 1.542-8.032; *P* < 0.001), indicating a persistent strong association. Further adjustment for confounders (smoking, alcohol consumption, diabetes mellitus, family histories of hypertension, diabetes mellitus, CAD, UA, and TG) yielded ORs of 2.923 (95% CI: 1.283-6.656; *P* < 0.001) in Model 3 and 3.086 (95% CI: 1.333-7.142; *P* < 0.001) in Model 4. Even after comprehensive multivariable adjustment, apoB/apoA-1 remained the biomarker most strongly associated with an increased PCAD risk compared to other inflammatory markers. In conclusion, MHR, NLR, apoB/apoA-1, and LDL-c/HDL-c were independent risk factors for PCAD, with apoB/apoA-1 demonstrating the strongest association.

### Correlations of apoB/apoA-1 with relevant parameters

Spearman’s correlation analysis showed that the apoB/apoA-1 was positively correlated with neutrophil, lymphocyte, monocyte, UA, TG, and LDL-c (*P* < 0.001), while it was negatively correlated with age and HDL-c (*P* < 0.001) (**Table 2**).

**Table 2:** Relationships of apoB/apoA-1with relevant parameters.

Variables	rs	*P* value
Age (years)	-0.124	<0.001
Neutrophil (×10^9^/L)	0.338	<0.001
Lymphocyte (×10^9^)/L)	0.097	0.002
Monocyte (×10^9^/L)	0.196	<0.001
UA (μmol/L)	0.203	<0.001
TG (mmol/L)	0.238	<0.001
LDL-c (mmol/L)	0.382	<0.001
HDL-c (mmol/L)	-0.243	<0.001

LDL-c, Low-density lipoprotein cholesterol; HDL-c, High-density lipoprotein cholesterol; UA, Uric acid; TG, Triglyceride; rs, Spearman’s rank correlation coefficient.

### Evaluation of the diagnostic efficacy of inflammatory markers for PCAD

**Figure 4:**
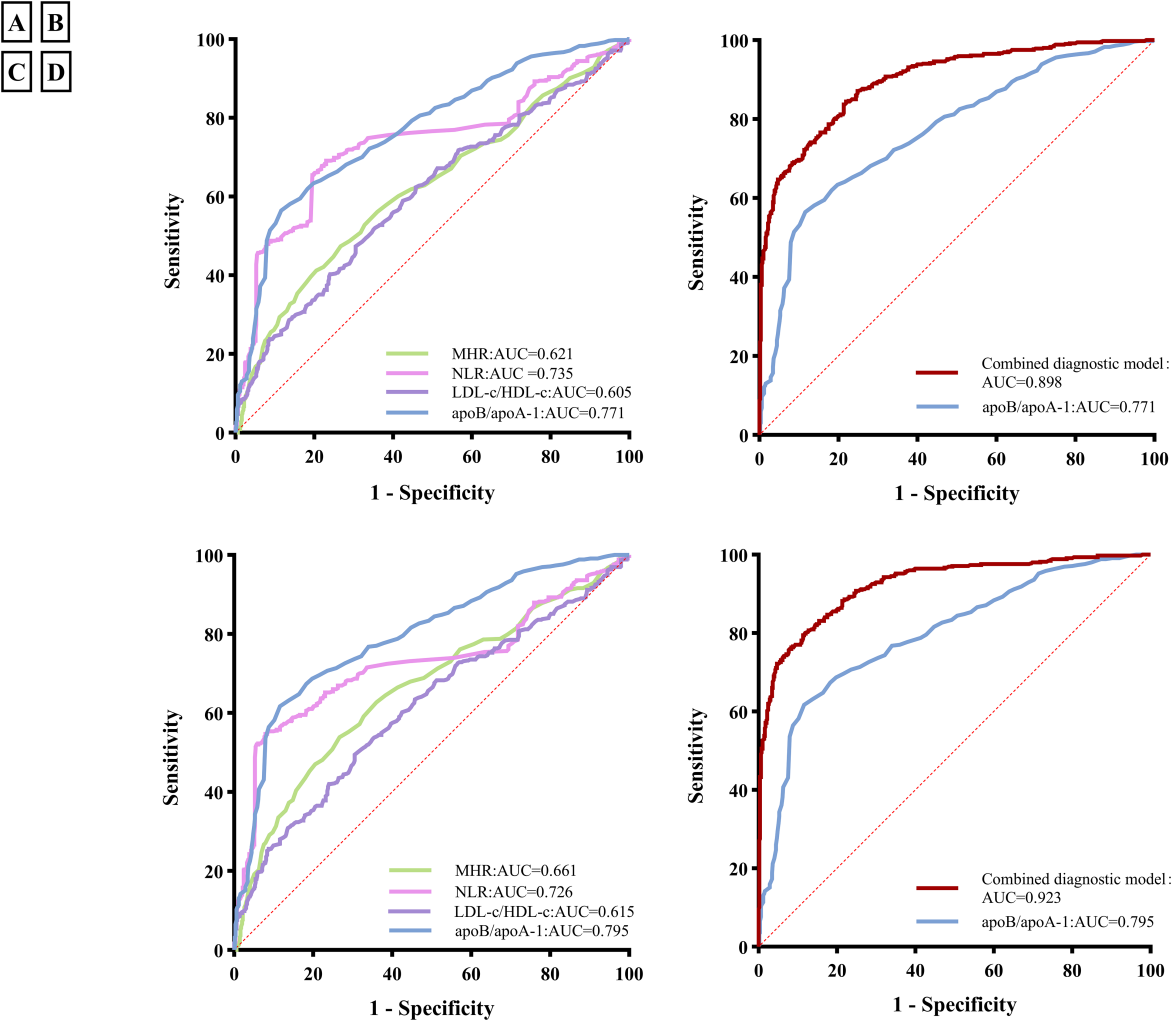
Receiver operating characteristic (ROC) curves of inflammatory markers and the combined diagnostic model in premature coronary artery disease (PCAD); **(A)** ROC curves of four inflammatory markers for PCAD; **(B)** ROC curves of the combined diagnosis model for PCAD; **(C)** ROC curves of inflammatory markers for acute coronary syndrome(ACS) in PCAD; **(D)** ROC curves of the combined diagnosis model for ACS in PCAD. MHR, Monocyte-to-high-density lipoprotein cholesterol ratio; NLR, Neutrophil-to-lymphocyte ratio; ApoB/apoA-1, Apolipoprotein B to apolipoprotein A-1 ratio; LDL-c/HDL-c, Low-density lipoprotein cholesterol to high-density lipoprotein cholesterol ratio; AUC, Area Under the Curve; the combined diagnostic model combining the apoB/apoA-1 with neutrophil, lymphocyte, monocyte, uric acid, triglycerides, HDL-c, Age, sex, smoking, alcohol consumption, diabetes mellitus and family histories of hypertension, diabetes mellitus, and CAD.

To evaluate the diagnostic ability of inflammatory markers for PCAD risk, ROC analysis was performed. As shown in **Figure 4A**, the Area Under the Curve (AUC) values for the four inflammatory markers were as follows: MHR 0.621 (95% CI: 0.587-0.656), NLR 0.735 (95% CI: 0.703-0.766), LDL-c/HDL-c 0.605 (95% CI: 0.570-0.640), and apoB/apoA-1 0.771 (95% CI: 0.742-0.799), respectively (all *P* < 0.001). These results indicate that apoB/apoA-1 had higher diagnostic value for PCAD than the other biomarkers.

The cutoff value of the apoB/apoA-1 was 0.824 with a sensitivity of 56.8% and a specificity of 88.4%. To further evaluate the diagnostic performance of the apoB/apoA-1 combined with serum indicators, the optimal diagnostic model was explored. As illustrated in **Figure 4B**, the combined diagnostic model combining the apoB/apoA-1 with neutrophil, lymphocyte, monocyte, UA, TG, HDL-c, Age, sex, smoking, alcohol consumption, diabetes mellitus and family histories of hypertension, diabetes mellitus, and CAD, had the highest AUC value of 0.898 (95% CI: 0.880-0.917) with a sensitivity of 84.50% and a specificity of 78.0%.

### Evaluation of the diagnostic efficacy of inflammatory markers for ACS in PCAD

To evaluate the diagnostic ability of inflammatory markers for ACS in PCAD patients, we stratified the PCAD cohort into: a stable angina group (n=64) and an ACS group (comprising 163 unstable angina and 294 myocardial infarction cases). The diagnostic performance of the biomarker model for ACS within this PCAD population was then analyzed. As shown in **Figure 4C**, the AUCs of the four inflammatory markers were as follows: MHR 0.661 (95%CI:0.626-0.696), NLR 0.726 (95%CI:0.692-0.760), LDL-c/HDL-c 0.615 (95%CI:0.579-0.651), and apoB/apoA-1 0.795 (95%CI:0.766-0.824), respectively (all *P* < 0.001). ApoB/apoA-1 demonstrated higher diagnostic value for ACS in PCAD than other biomarkers.

The cutoff value of the apoB/apoA-1 was 0.824 with a sensitivity of 61.9% and a specificity of 88.4%. To further evaluate the diagnostic performance of the apoB/apoA-1 combined with serum indicators, the optimal diagnostic model was explored. As illustrated in **Figure 4D**, the combined diagnostic model combining the apoB/apoA-1 with neutrophil, lymphocyte, monocyte, UA, TG, HDL-c, age, sex, smoking, alcohol consumption, diabetes mellitus, and family histories of hypertension, diabetes mellitus, and CAD, had the highest AUC value of 0.923 (95% CI: 0.906-0.940) with a sensitivity of 75.9% and a specificity of 92.3%.

## Discussion

CAD is increasingly affecting younger populations. With changes in lifestyle, the increased prevalence of metabolic diseases, and shifts in exposure patterns to risk factors, the characteristics and risk factors of CAD onset have also changed (31, 32). The risk factors for PCAD also differ from those for MCAD (33). CAD is a chronic inflammatory disease characterized by coronary artery stenosis and remodeling, resulting from the interaction of genetic and environmental factors (34, 35). Over the past two decades, the perspective on atherosclerosis has gradually shifted towards it being a lipid-driven, chronic, low-grade inflammatory disease of the arterial wall. Inflammatory factors and lipid factors are involved in its occurrence, progression, and prognosis (36). Therefore, in this study, we investigated the correlation between PCAD and inflammatory/lipid ratios. We found that MHR, NLR, apoB/apoA-1, and LDL-c/HDL-c all demonstrated diagnostic value for PCAD. Specifically, the apoB/apoA-1 ratio demonstrated the greatest diagnostic value. Its diagnostic power was further enhanced when combined with clinical risk factors. Furthermore, this diagnostic value was particularly pronounced in the ACS subgroup.

Our study showed that the PCAD group was younger than the angiographically normal control group. This age difference may be related to the higher proportion of males in the PCAD group, as the age inclusion criteria for males were lower than those for females. Furthermore, the younger age of the PCAD group better reflects its premature onset characteristics. This is because numerous studies have demonstrated that the onset of CAD and many of its risk factors are positively correlated with age (i.e., older age is associated with higher disease risk, more risk factors, and greater severity) (37–40). Paradoxically, the PCAD group, being the diseased group, was younger than the angiographically normal control group. This further underscores that the PCAD group possesses pathogenic factors that defy age-related distribution patterns compared to the angiographically normal controls. This paradox will further emphasize the significance of abnormal findings in the subsequent comparison of risk factors between the PCAD group and the angiographically normal control group.

Our study found that the PCAD group had a higher proportion of smokers, alcohol consumers, individuals with diabetes, and individuals with a family history of hypertension, diabetes, and CAD compared to the control group. This aligns with the established clinical understanding of CAD risk factors (34). The higher prevalence of family history of hypertension and diabetes likely involves complex interactions between genetic factors and environmental influences (41–43). Notably, no significant difference in the proportion of hypertension itself was observed between the two groups in this study. This finding is inconsistent with the widely accepted concept that hypertension is a causative factor for CAD, and with the majority of studies reporting a higher prevalence of hypertension among CAD patients (44, 45). This discrepancy may be related to regional variations in the prevalence of hypertension. Alternatively, it could be attributed to the relatively preserved cardiovascular status in younger patients, including their greater compensatory capacity to resist adverse stimuli, enhanced ability to clear inflammatory mediators, and stronger vascular barrier function. Furthermore, as a chronic disease, hypertension requires a longer duration to exert its pathogenic effects on the cardiovascular system. Therefore, hypertension itself may have limited reference value for predicting PCAD onset. Nevertheless, strict blood pressure control remains crucial for young patients, as the risk of developing CAD increases with advancing age and prolonged duration of hypertension.

Regarding laboratory parameters in PCAD, research is relatively scarce. This study found that PCAD patients had elevated levels of Neutrophil, Lymphocyte, Monocyte, UA, TG, and apoB, while levels of apoA-1 and HDL-c were decreased. These alterations align with the characteristic features of CAD pathogenesis and are even more pronounced in PCAD (16). Notably, PLR showed no significant difference between groups (**Table 1**), and LMR failed to demonstrate independent association with PCAD in regression models (**Figure 3**). This apparent non-significance may reflect pathophysiological specificity: PLR’s focus on platelet activation aligns poorly with the chronic monocyte-driven inflammation of young-onset PCAD, while LMR’s signal is attenuated by compensatory lymphocyte preservation and monocyte subset differentiation better captured by ratios like MHR. Furthermore, in this study, PLT count, TC, and LDL-c levels in the PCAD group showed no significant difference compared to the angiographically normal control group. This finding differs from the results of Duran M’s study (46). Duran M’s research indicated that young patients (< 60 years old with stable angina) had lower PLT counts and higher TC and LDL-c levels compared to angiographically normal controls, and identified LDL-c as an independent risk factor for young patients. The discrepancy between the two studies may be related to differences in the inclusion criteria of the study populations and ethnic variations. Differences in lifestyle and dietary habits associated with geographical regions could also have played a role. Consequently, the results of this study only suggest that PLT count, TC, and LDL-c may contribute less to PCAD development in the Chinese population of the Zunyi region.

Research on diagnostic models specifically for PCAD is relatively scarce, and there is no established mainstream consensus; most findings are extrapolated from studies on CAD. This study found that the apoB/apoA-1 diagnostic model demonstrated the greatest value for predicting PCAD. This diagnostic value was further enhanced when combined with certain clinical risk factors and was particularly pronounced within the ACS subgroup.

ApoB is the core structural protein of atherogenic lipoproteins (such as Lp (a), LDL, VLDL), and atherosclerosis is the underlying cause of heart attacks. The infiltration and retention of apoB-containing lipoproteins within the arterial wall constitute a key initiating event that triggers inflammation and promotes the development of atherosclerosis. Following penetration through damaged endothelium, apoB-containing lipoproteins become oxidized to form ox-LDL, which activates macrophage scavenger receptors (e.g., CD36), promoting foam cell formation. ApoB also activates the NLRP3 inflammasome, leading to the release of IL-1β and IL-18, thereby intensifying the inflammatory microenvironment within plaques. Furthermore, apoB levels are associated with an enlarging lipid core and thinning fibrous cap, increasing the risk of plaque rupture (47, 48). Consequently, some researchers posit that apoB is a more accurate marker of cardiovascular risk than LDL-c or non-HDL-c (49). ApoA-1 is the primary protein component of HDL-c, well-known for its roles in regulating cholesterol transport, modulating inflammation and immune responses, and preventing atherosclerosis. ApoA-1 facilitates cholesterol efflux from cells via ABCA1/ABCG1 transporters, reducing lipid deposition in the vascular wall. ApoA-1 can inhibit the NF-κB pathway and macrophage polarization, thereby attenuating plaque inflammation. It also enhances mitophagy through the JAK2/STAT3 pathway, improving oxidative stress (50, 51). In summary, apoB drives atherosclerotic progression through multiple pathways, including lipid retention, inflammation activation, plaque destabilization, and genetic regulation, whereas ApoA-1 exerts opposing protective effects. Both are critical factors influencing atherosclerosis in its early stages. The apoB/apoA-1 ratio model integrates the diagnostic capabilities of both markers and can serve as a diagnostic model for PCAD.

In recent years, numerous innovative diagnostic approaches have emerged for PCAD, including but not limited to machine learning algorithms (52), IL-6 gene polymorphism screening (53) and electrochemical biosensors (54). While demonstrating promising diagnostic efficacy, their clinical implementation nevertheless faces substantial barriers. These include prohibitive per-test costs ($50–$1000), dependency on specialized instrumentation, and operational complexities that hinder integration into routine screening protocols. By contrast, the apoB/apoA-1 ratio model leverages existing lipid profiling infrastructure, utilizing validated thresholds to enable cost-effective risk stratification (<$5 per test). This positions it as a pragmatic solution for large-scale screening of non-traditional risk cohorts, particularly individuals with isolated familial predisposition. The findings of this study therefore establish a framework for incorporating coronary risk assessment into standard physical examinations, with the biomarker models investigated offering exceptional economic viability and clinical deployability through their accessibility via routine laboratory assays.

As a cross-sectional analysis, this research primarily establishes the diagnostic utility of inflammatory ratios for identifying existing PCAD. While the developed models demonstrate strong discriminatory capacity within this context, their true predictive capability for incident PCAD remains to be validated. Robust validation of this predictive capability requires future prospective cohort studies incorporating pre-diagnosis biomarker measurements and longitudinal follow-up prior to disease onset. Furthermore, the single-center retrospective design inherently limits the generalizability of our findings—confounding factors such as genetic predispositions (e.g., lipid metabolism polymorphisms unique to the Southwestern Han population), lifestyle patterns (high-salt and high-fat diets, capsaicin intake), environmental exposures (trace element distribution in karst topography), regional patient characteristics, healthcare practice variations, and selection biases may influence the conclusions.

Therefore, although these initial results highlight the significant potential of apoB/apoA-1 and its combined model for diagnosis, their clinical translation necessitates validation in multi-center prospective cohorts with predefined endpoints and external datasets to confirm generalizability and establish real-world clinical utility. Additionally, while we investigated several established inflammatory ratios and traditional risk factors, the range of potential predictors examined could be expanded and refined in future research, and a larger sample size would enhance the statistical power and stability of the models, particularly for subgroup analyses.

Future research should aim to: conduct multi-center prospective studies with pre-diagnostic biomarker assessment to validate the predictive value of these ratios for incident PCAD; incorporate external validation cohorts; explore the integration of a broader spectrum of novel biomarkers and risk factors; and investigate the cost-effectiveness and clinical impact of implementing apoB/apoA-1 screening in routine health assessments for early PCAD detection.

## Conclusions

The apoB/apoA-1 outperformed MHR, NLR, and LDL-c/HDL-c as an inflammatory biomarker in PCAD. Its diagnostic capacity was notably enhanced in ACS subgroups. A comprehensive model combining apoB/apoA-1 with traditional risk factors demonstrated exceptional accuracy. Incorporating this biomarker into routine screening protocols could significantly strengthen preventive strategies.

## Data Availability

The raw data supporting the conclusions of this article will be made available by the authors, without undue reservation.
